# New Late Permian tectonic model for South Africa’s Karoo Basin: foreland tectonics and climate change before the end-Permian crisis

**DOI:** 10.1038/s41598-017-09853-3

**Published:** 2017-09-07

**Authors:** Pia A. Viglietti, Bruce S. Rubidge, Roger M. H. Smith

**Affiliations:** 1 Evolutionary Studies Institute, University of the Witwatersrand, Johannesburg, Private Bag 3 Wits 2050, South Africa; 2 School for Geosciences, University of the Witwatersrand, Johannesburg, Private Bag 3 Wits 2050, South Africa; 3 Iziko South African Museum of Cape Town, P.O. Box 61, Cape Town, 8000 South Africa

## Abstract

Late Permian Karoo Basin tectonics in South Africa are reflected as two fining-upward megacycles in the Balfour and upper Teekloof formations. Foreland tectonics are used to explain the cyclic nature and distribution of sedimentation, caused by phases of loading and unloading in the southern source areas adjacent to the basin. New data supports this model, and identifies potential climatic effects on the tectonic regime. Diachronous second-order subaerial unconformities (SU) are identified at the base and top of the Balfour Formation. One third-order SU identified coincides ﻿with﻿ a faunal turnover which could be related to the Permo-Triassic mass extinction (PTME). The SU are traced, for the first time, to the western portion of the basin (upper Teekloof Formation). Their age determinations support the foreland basin model as they coincide with dated paroxysms. A condensed distal (northern) stratigraphic record is additional support for this tectonic regime because orogenic loading and unloading throughout the basin was not equally distributed, nor was it in-phase. This resulted in more frequent non-deposition with increased distance from the tectonically active source. Refining basin dynamics allows us to distinguish between tectonic and climatic effects and how they have influenced ancient ecosystems and sedimentation through time.

## Introduction

The Karoo Basin of South Africa represented a large depocenter situated in southern Gondwana supported by the Kaapvaal Craton in the northeast and the Namaqua-Natal Metamorphic Belt (NNMB) in the southwest^1, 2^. Deposition and accumulation in the Karoo Basin began with the Dwyka Group (320–280 Ma)^3^, followed by the Ecca, Beaufort, and Stormberg groups respectively, finally terminating after more than 100 Million years of sedimentation with the igneous basaltic outpourings of the early Jurassic Drakensberg Group^1, 4–9^. Theories on the origin of accommodation in the Karoo Basin and distance from the Paleo-Pacific subduction zone (>1500 km)^10^ and Gondwanan Mobile Belt (GMB) include, activation of the Southern Cape Conductive Belt, a crustal geophysical anomaly^11^, fault-controlled subsidence^2, 12^, and continent-continent collision with south dipping subduction zone^13^. Despite disagreements, most workers recognize foreland basin tectonics with a north dipping subduction zone in the Karoo basin and it is only the timing of the onset of foreland tectonics that has been seriously disputed. It is now clear that by the time of deposition of the Dwyka Group, foreland tectonics were already operating^3, 14–16^. Thus the Karoo Basin was most likely a foreland basin from its formation and these conditions continued until its termination at the end of the Stormberg Group^1, 17^. Additionally, northward thinning of strata, condensed, diachronous, and missing lithostratigraphic units away from the source area reflects many characteristics of modern foreland systems^18–22^. Thus, foreland tectonics, with dynamic subsidence resulting from orogenic loading by flat-slab subduction^23, 24^, is currently the most popular explanation for the generation of accommodation in the main Karoo Basin^1, 3, 5, 10, 16, 25–34^.

Flexural tectonics partitioned the Karoo Basin into the foredeep, forebulge, and backbulge flexural provinces with a dominantly southerly sediment source area^1, 4^. Orogenic loading and unloading in the GMB changed the forebulge and foredeep’s positions, and this resulted in deposition in the proximal basin in the south during loading and distal regions of the Karoo Basin in the north during unloading. Where a time lag occurred between orogenic loading and basinal subsidence^24, 26^ the upper and lower boundaries of lithostratigraphic units are diachronous, and have proximal and distal equivalents. Thus, many of the upper and lower boundaries of lithostratigraphic groups are diachronous or have proximal and distal equivalents^1, 4, 35^. This is reflected in the geologic record as the preservation of large-scale (second-order) fining upward depositional sequences bounded by subaerial unconformities (SU)^1, 36, 37^. Thus the SU in this study were identified by relatively abrupt changes in lithology (e.g. renewed fining upward cycles), mean change in paleocurrent direction, changes in fluvial style (e.g. from high to low sinuosity), and rarely by palaeoclimatic changes and faunal turnover^38^.

Modern and ancient foreland basins like the Karoo Basin are bounded by tectonically active source areas. Episodic tectonic activity results in the rejuvenation of fluvial systems leading to deposition of coarser sediment, and cessation in tectonic activity results in an overall fining upward sequence^37^. These cycles are recognized in the Beaufort Group^39^ by fluvial systems that are characterized by an initial pulse of high energy fluvial transport (e.g. sandstone-rich lithological unit), often underlain by an SU, followed by increasingly finer grained sediment as tectonic activity ceased^1^ and river gradients declined. Similarly, this cyclicity is used to identify the position of SUs in Upper Cretaceous fluvial deposits in southern Utah^40, 41^.

**Figure 1:**
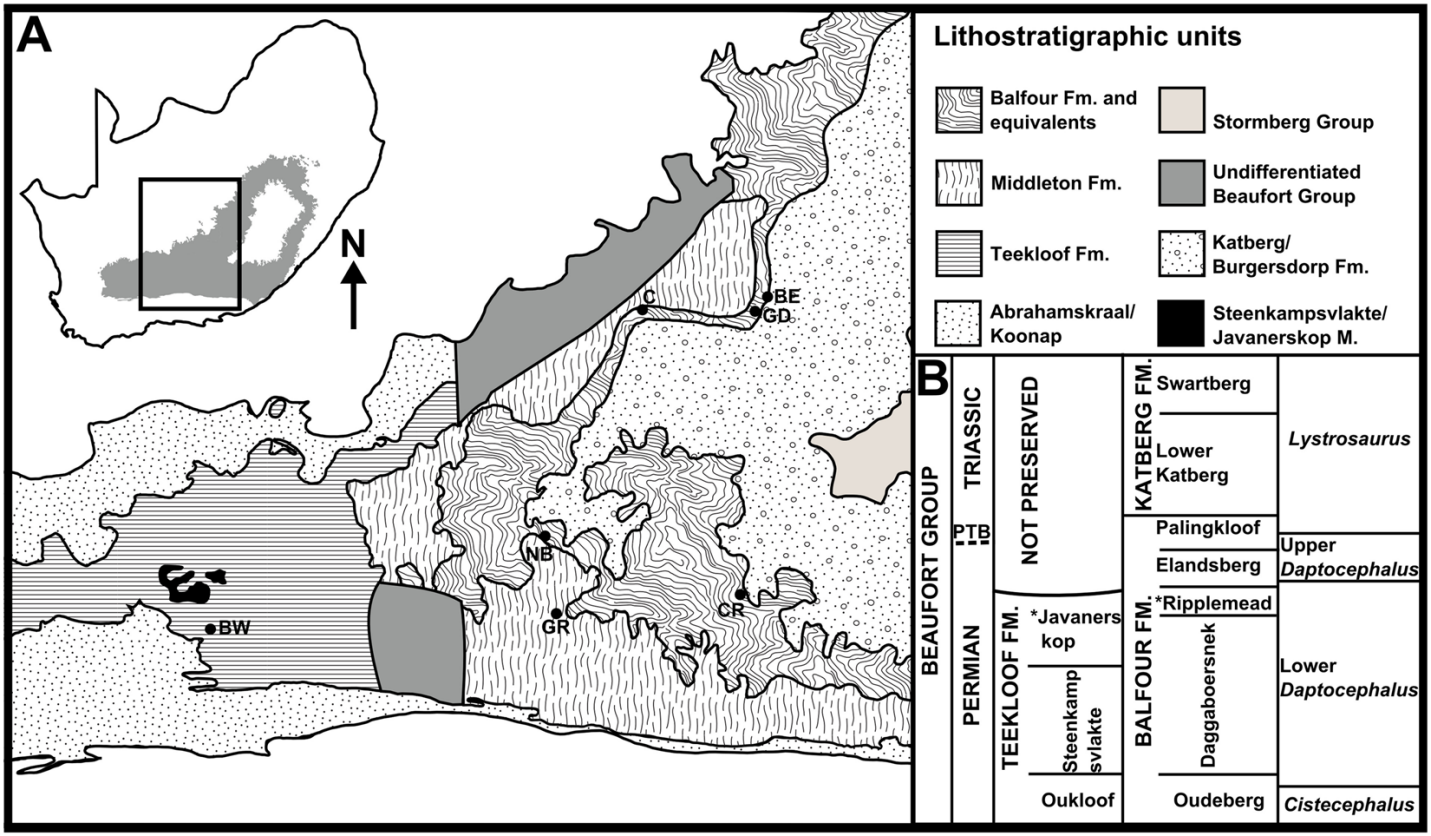
(**A**) Map of South Africa’s Karoo Basin showing local geology and position of the field sites. The field sites are near Gariep Dam (GD), Cradock (CR), Nieu Bethesda (NB), and Beaufort West (BW). (**B**) Current lithostratigraphic subdivisions of strata correlated to the *Daptocephalus* and *Lystrosaurus* assemblage zones. All cartographic information was reported by PAV and by referring to written literature cited from^35, 66–68^. All this information was reproduced by PAV using Inkscape (vers. 0.91, https://inkscape.org/en/release/0.91/platforms/).

The most recent sequence stratigraphic research on the Beaufort Group’s Balfour Formation identified six, unconformity-bound third-order depositional sequences within a 2150 m interval^36^, which are the result of smaller-scale orogenic events. Recently, the Late Permian Beaufort Group stratigraphy has been revised^42, 43^ (Fig. 1). This study reviews the tectonic setting of the Late Permian Karoo Basin and uses sequence stratigraphy to provide an updated basin development model. It also discusses the implications of our increased understanding of Late Permian Karoo Basin dynamics and how to recognize the climatic overprint on foreland basin sequences.

## Results

**Figure 2:**
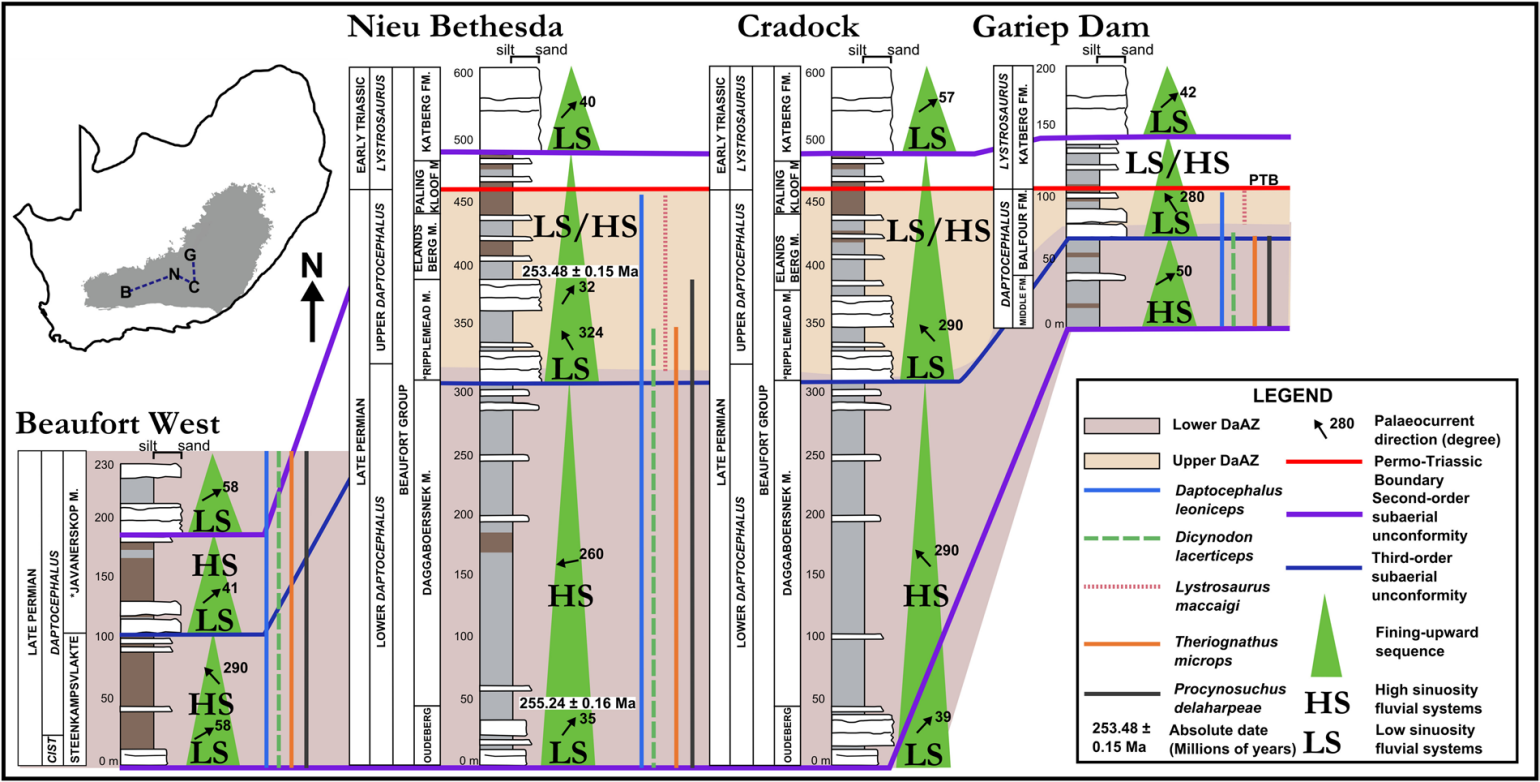
Composite sections created for the field sites of this study. Faunal ranges of *Daptocephalus* Assemblage Zone index taxa are shown in conjunction with stratigraphic position of low and high sinuosity fluvial systems, second-order and third-order subaerial unconformities (SU), palaeocurrents, and absolute dates. The migration of this SU east and northwards fits with observations made from faunal ranges that uppermost Permian stratigraphic succession is more complete in the south than in the north of the basin. All vertical and cartographic information was recorded and reproduced by PAV and reproduced using Inkscape (vers. 0.91, https://inkscape.org/en/release/0.91/platforms/).

**Figure 3:**
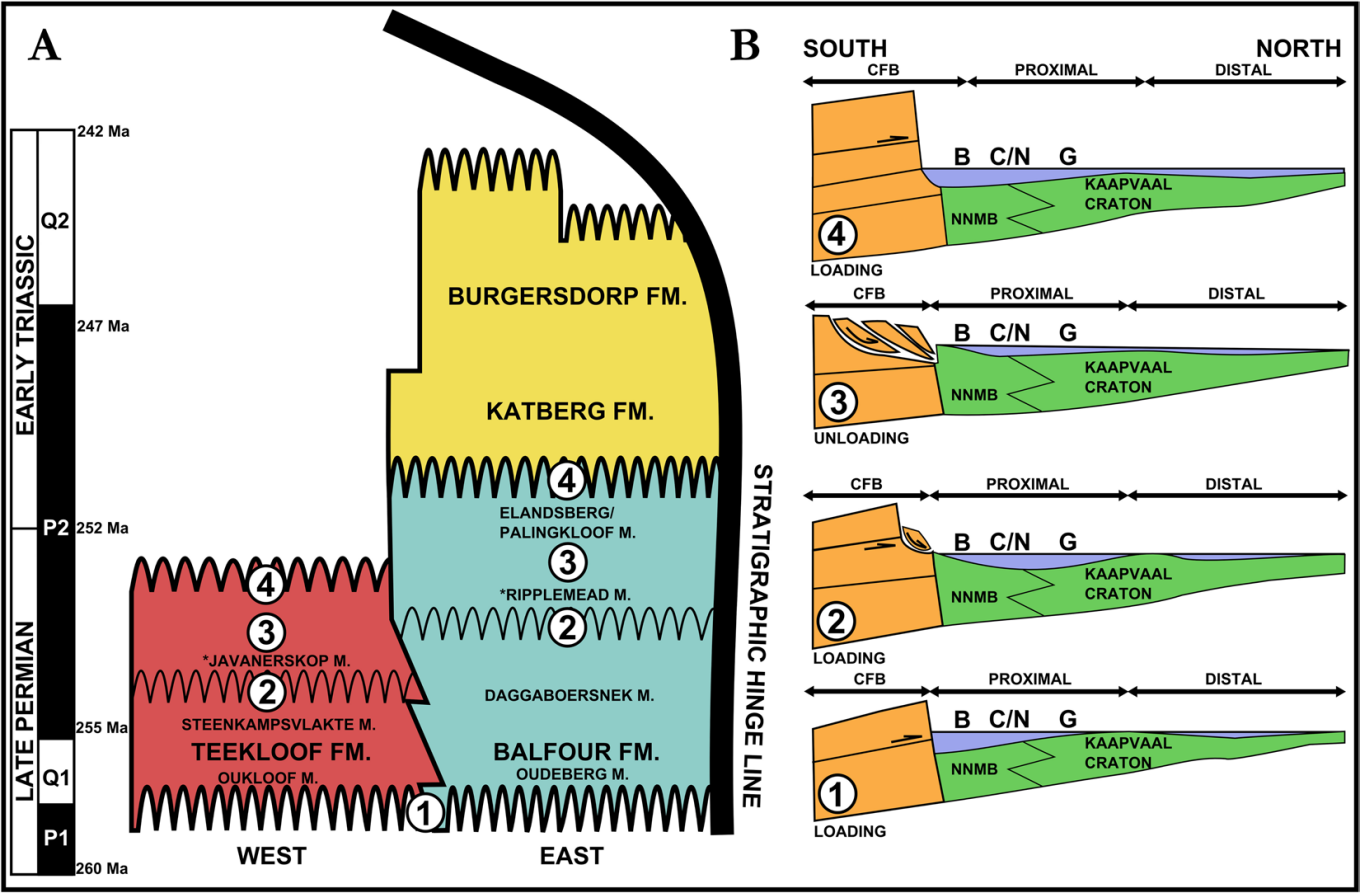
(**A**) Sequence stratigraphic framework for the Late Permian-Lower Triassic Karoo Basin in relationship to new paroxysm dates^61^ and the stratigraphic hinge line^1^. Thick wavy lines depict the second-order sequence boundaries (SU). Thinner wavy lines indicate a third-order SU. (**B**) Schematic models for the evolution of the Karoo Basin during *Daptocephalus* Assemblage Zone times in the Late Permian to Lower Triassic. Letters represent field sites Beaufort West (B), Cradock (C), Nieu Bethesda (N), Gariep Dam (G). Proximal represents the entire foredeep in this diagram and distal is the back bulge. The Cape Fold Belt (CFB) represents the source of sediment and tectonic activity south of the basin. Numbered 1–4, the basin schematics depict changes in orogenic loading and unloading and the effect this had creation of accommodation in the Karoo foreland basin. All information was reported and reproduced by PAV using Inkscape (vers. 0.91, https://inkscape.org/en/release/0.91/platforms/).

Composite sections of the four field sites (Fig. 2), and a suggested basin model for the Late Permian Karoo Basin (Fig. 3) present the results. The composite sections document two fining-upward sequences in the Balfour and upper Teekloof formations. Our study also identifies three subaerial unconformities (SU) using the criteria outlined previously (Fig. 2). Two second-order SUs are present at the base and top of the Balfour Formation^1^ and one third-order SU is noted in the middle of the Balfour Formation, which only attains a maximum thickness of 513 m. This contradicts previous observations on the sequence stratigraphy of the Balfour Formation, whose six third-order SU over a 2150 m stratigraphic interval^36^ are based only on estimated Late Permian lithostratigraphic thicknesses^44, 45^. Our study extends the second-order SU at the base of the Balfour Formation west to the base of the Oukloof Member (upper Teekloof Formation) and northwards towards the basin’s forebulge (Figs 2 and 3), updates biostratigraphic ranges, and absolute dates^46, 47^ show the SU are diachronous. *Daptocephalus* Assemblage Zone tetrapod fauna provide further evidence for out-of-phase sedimentation because they occur in different lithostratigraphic units in different parts of the foreland system. From absolute dates which constrain their stratigraphic distribution in the eastern basin, the conclusion can be made that the SU boundaries are older in the western part of the basin (Fig. 2).

## Discussion

Although some of the SU presented in this study have been observed in the Karoo Basin by previous workers^1, 34, 36, 48–50^, they were interpreted as having a purely tectonic origin as uplift leads to increased stream gradients, sediment input, and accommodation proximal to the source area^1, 36^. However, climatic, and tectonic signatures are shown to overprint one another during the onset and aftermath of the Permo-Triassic mass extinction event (PTME)^51–57^. This study provides some evidence that climatic changes associated with the PTME were occurring much lower in the stratigraphy than previously documented. Thus it is concluded the third-order tectonic SU could be overprinted by these palaeoclimatic changes. This is supported by a newly identified faunal turnover and abundance changes marked by the appearance of *Lystrosaurus maccaigi* and disappearance of *Dicynodon lacerticeps*, *Theriognathus microps*, and *Procynosuchus delaharpeae* in this SU. Palaeoclimatic changes at the point of the third-order SU in the *Daptocephalus* Assemblage Zone are identified by a decrease in the occurrence of subaqueous environments in the overbank facies^42^. In addition, distal attenuation of the Balfour Formation towards the north indicates there was more accommodation for sediment proximally, and point to more non-deposition distally during the Late Permian^42, 43^. Although this faunal turnover is not identified at the base of the SU in the Beaufort West field site, it implies that the third-order SU is diachronous. Diachroneity has been identified at major lithostratigraphic boundaries of the Karoo Supergroup, such as the Ecca-Beaufort contact, Katberg-Burgersdorp contact, and Burgersdorp-Molteno contact^1, 58–60^ and is evidence of tectonic influences controlling sediment distribution in the foredeep.

## New Tectonic Model

All evidence collected indicates that cyclic, out-of-phase sedimentation in the Karoo Basin during the Late Permian resulted from a strong tectonic influence south of the basin which was also the source of clastic material. Our depositional model refines third-order detail in addition to the mostly first and second-order framework of the Karoo Basin sedimentary fill^1, 36^. Each second or third-order sequence correlates to an orogenic cycle of thrusting followed by unloading. New paroxysm dates^61^ fall within the dates of known active orogenic loading.

It is interesting to note that although out-of-phase, the western and eastern basin (Teekloof and Balfour formations) show similar lithostratigraphic sequences and it is clear they were influenced by similar tectonic and climatic events in the basin (Fig. 3). The western and eastern sectors of the Karoo Basin could represent two distinct Distributive Fluvial Systems (DFS)^62, 63^ and the geophysically- defined “Willowmore arch”^64^, which is interpreted as a palaeotopographic high, was present between these two parts of the basin. This may explain some differences between the two parts of the basin but essentially, they are reflecting similar tectonic events in the foreland system defined by fairly repetitive, and roughly synchronous, depositional cycles^37^. This also entertains the possibility of alternative controls on subsidence that caused the basin to contain down-warped depressions and upward arches formed by the interplay of the foreland compressional tectonics and local rheology and structural integrity of the basement rocks. The Kaapvaal Craton situated below the distal basin would have been more rigid and buoyant than the Namaqua-Natal Metamorphic Belt which comprises the basement rocks of the proximal foredeep and back bulge. Structural anomalies could have controlled tectonics locally^2, 65^, and may have contributed to the out-of-phase deposition created by flexural tectonics of the foreland system. This study’s basin model has four stages which explain the inferred tectonic setting of the Late Permian Karoo Basin (Fig. 3).

Stage 1 Follows orogenic uplift in the Gondwanide Mobile Belt with the onset of sedimentation of the Balfour in the southeast and upper Teekloof formations in the southwest of the basin which began at the base of the first second-order SU and represents the beginning of a fining-upward cycle deposited by low sinuosity rivers (the eastern Oudeberg and western Oukloof members). Distally, there was non-deposition as no lithostratigraphic equivalent is documented close to the forebulge. Once sediment supply became less than available accommodation, the sequence began to fine-upward, depositing argillaceous material by high sinuosity rivers (the eastern Daggaboersnek and western Steenkampsvlakte members).

Another minor orogenic loading event initiated stage 2, resulting in the out-of-phase deposition of the arenaceous Javanerskop member in the west and Ripplemead member in the east above the third-order SU. On current biostratigraphic evidence^43^, the Javanerskop member appears to be slightly older than the Ripplemead member and is present only in the south east and central Karoo Basin. Coincidently, the decrease in sinuosity of the fluvial systems leads to reduced overbank preservation, and disappearance of lacustrine systems.

Stage 3 is an unloading phase resulting in distal progradation of sediment when accommodation became available in the distal foredeep. This explains the attenuated but similar stratigraphy. The third-order sequence began to fine upwards, terminating in the argillaceous Elandsberg and Palingkloof members (Figs 2 and 3). This sequence was overprinted by climatic changes during the onset of the PTME^55^ as indicated by paleoclimatic changes and a newly identified faunal turnover. Stage 4 marks the beginning of deposition of the early Triassic lower Katberg Formation during a new orogenic loading phase.

## Conclusions

Flexural tectonics imposed phases of orogenic loading and unloading during the Late Permian Karoo Basin. This was the major control on the distribution of accommodation in the foredeep of the Karoo Basin as demonstrated by northward thinning wedge-shaped deposition of strata and cyclic sedimentation bounded by two second-order, and one third-order subaerial unconformities (SU)^37, 40, 41, 62, 63^. Foreland tectonics meant that progradation of sediment northwards (distal foredeep) during orogenic loading and unloading was not in phase with the south (proximal foredeep) which resulted in an incomplete and attenuated stratigraphic record to the north, and diachronous SU between the west and eastern parts of the basin.

Throughout the depositional cycle of the Late Permian Beaufort Group, little evidence for significant climatic change has been documented^1, 34, 36^, but tectonic and climatic signals are notoriously difficult to tease apart in the geologic record^27, 37^. The revised Late Permian stratigraphy and tectonics^42, 43^ indicate that there was some climatic change overprinting the tectonic signatures imposed by orogenic loading^1^ at least by the Upper *Daptocephalus* Assemblage Zone (DaAZ), and the third-order SU, as fauna began to go extinct or decrease in abundance^42, 43^. This new basin model, in combination with revised Late Permian stratigraphic investigations, tells us that environmental changes may have been occurring earlier than previously documented and they could be related to the effects of the PTME^55^. Thus, understanding ancient ecosystems has important implications for the understanding basin dynamics, especially in the Late Permian Karoo Basin where there is much evidence for a coeval terrestrial counterpart to the global Permo-Triassic mass extinction (PTME)^51, 53, 55–57^. There is still much debate concerning what role tectonics and climate played in this major biotic crisis, and studies such as this contribute to understanding this important event in Earth’s history.

## Methods and Materials

The study area encompassed the stratigraphic range of the *Daptocephalus* Assemblage Zone (Balfour and upper Teekloof formations). Four field sites were chosen near Cradock, Nieu Bethesda, Beaufort West, and Gariep Dam (Fig. 1). Numerous fossils were collected and stratigraphically recorded. Vertical sections measured compiled four composite sections, one for each field site. This was done to investigate Late Permian Karoo Basin litho, bio, and sequence stratigraphy. This research also required referral to research conducted by previous workers to corroborate findings^36, 54^.
